# Condom use and drug consumption in migrants: a systematic review

**DOI:** 10.1590/1980-220X-REEUSP-2023-0032en

**Published:** 2023-11-17

**Authors:** Cynthia Lizbeth Ruiz-Bugarin, Ulises López-Sánchez, Jesus Ramon Aranda-Ibarra, Carmen Ivette Hernández-Vergara, Jimenez Caro-Jocelyn, Mario Alberto Sánchez-Rojas, Anzony Arturo Cruz-González

**Affiliations:** 1 Universidad Autónoma de Baja California, Facultad de Ciencias de la Salud, Tijuana, México.; 2 Colegio de Profesionales de la Enfermería de Baja California AC, Tijuana, Baja California, México.

**Keywords:** Condoms, Substance-Related Disorders, Illicit Drugs, Transients and Migrants, Preservativos, Transtornos Relacionados ao Uso de Substâncias, Drogas Ilícitas, Migrantes, Condones, Trastornos Relacionados con Sustancias, Drogas Ilícitas, Migrantes

## Abstract

**Objective:**

To identify condom use and drug consumption in migrants, as well as the association between these variables.

**Method:**

A systematic search was carried out for articles published in Spanish and English (2017-2022), in PubMed, EBSCO, WEB of SCIENCE, Elsevier, Scielo, Redalyc, with eligible studies reporting on condom use and drug consumption, and their association.

**Results:**

The search strategy found 147 articles with the combination of terms and other sources. After excluding articles by title, abstract, and finding that they had the study variables, eight articles were included for qualitative analysis and only three met the criteria for quantitative analysis.

**Conclusion:**

Drug consumption favors inconsistent condom use, increasing the risk of acquiring an STI, and can lead to other mental health issues derived from the use of these substances.

## RESUMEN

**Objetivo:**

Identificar el uso del condón y el consumo de drogas en migrantes, así como la asociación entre estas variables.

**Método:**

Se realizó una búsqueda sistemática de artículos publicados en español e inglés (2017–2022), en PubMed, EBSCO, WEB of SCIENCE, Elsevier, Scielo, Redalyc, siendo que los estudios elegibles informaron sobre el uso del condón y el consumo de drogas, y su asociación

**Resultados:**

Con la estrategia de búsqueda se identificaron 147 artículos con la combinación de términos y de otras fuentes. Después de la eliminación de artículos por título, resumen, identificar que cuenten con las variables de estudio, se incluyeron ocho artículos para el análisis cualitativo y únicamente tres cumplieron con los criterios para el análisis cuantitativo.

**Conclusión:**

El consumo de drogas favorece el uso inconstante del condón, y esto incrementa el riesgo de adquirir alguna ITS, además de otras cuestiones de salud mental derivadas del consumo de estas sustancias.

## RESUMO

**Objetivo:**

Identificar o uso de preservativo e o consumo de drogas em migrantes, bem como a associação entre essas variáveis.

**Método:**

Foi realizada uma busca sistemática de artigos publicados em espanhol e inglês (2017-2022), em PubMed, EBSCO, WEB of SCIENCE, Elsevier, Scielo, Redalyc, com estudos elegíveis relatando o uso de preservativos e consumo de drogas, e sua associação.

**Resultados:**

Com a estratégia de busca foram identificados 147 artigos com a combinação de termos e outras fontes. Após eliminar os artigos por título, resumo e identificar que continham as variáveis do estudo, oito artigos foram incluídos para análise qualitativa e apenas três atenderam aos critérios para análise quantitativa.

**Conclusão:**

O consumo de drogas estimula o uso inconsistente do preservativo, o que aumenta o risco de aquisição de uma IST, além de outros problemas de saúde mental decorrentes do consumo dessas substâncias.

## INTRODUCTION

Currently, both sexually transmitted infections (STIs) and addictions are a major public health problem. The World Health Organization reports that there are 38.4 million people living with the Human Immunodeficiency Virus and Acquired Immunodeficiency Syndrome (HIV/AIDS)^(1)^ worldwide, of which approximately 2.1 million live in Latin America^(2)^. The spread of communicable diseases has been associated with population mobility, which includes migrants, who are three times more likely to acquire HIV/AIDS^(3)^ compared to other population groups, as migration allows that a greater number of people interact with each other, having multiple and recurrent unprotected sexual relations, and use drugs and alcohol, therefore increasing the likelihood of transmission of infectious diseases, including people who are deported^(4)^.

In addition to risky sexual behaviors, migration can also affect the type and frequency of substance use. Drug consumption is a phenomenon that has been increasing, year after year. It is estimated that, in 2020, of the world population aged between 15 and 64, 284 million people consumed some type of drug. Regarding the consumption of illicit drugs worldwide, 209 million people used cannabis in 2020, making it the most consumed drug^(5)^.

Today, sexual health and addiction issues are associated with population mobility, ignorance, low perception of the risk of acquiring sexually transmitted infections, and the use of injectable drugs. The literature reports that migrants present risky sexual behaviors, derived from individual, psychological, sociocultural, cognitive, and motivational factors, which increase the risk of acquiring HIV, due to all the vulnerabilities that migrants undergo during their journey^(6)^. Finding the relationship between condom use and drug consumption in migrants can facilitate the identification of the dimensions of knowledge that are important to base more effective cognitive-behavioral interventions.

In 2021, a literature review was carried out in a period of 10 years (2011 to 2021), which found that the main risk factors for acquiring an STI are: sexual intercourse without the use of a condom, multiple sexual partners, younger age of initiation of sexual activity, HIV infection, low educational level, factors related to the route of transmission, bacterial vaginosis, migration, alcohol consumption, travel, history of childhood sexual abuse, and others, such as lack of circumcision, intravenous drug use, prostitution, and lack of knowledge^(7)^. Another study carried out on drug consumption and HIV/AIDS in Central America presents different models and theories that try to explain the behavior issues, as well as risk levels^(8)^.

For the purposes of this study, condom use is defined as the reported frequency of condom use in the migrant population; drug consumption, as the use of any chemical substance, by any route of administration, which can alter the functioning of the central nervous system, and therefore the behavior of the migrant, causing dependence. A review of the literature was carried out to identify condom use and drug consumption in migrants, as well as the association between these variables, considering primary studies published in the last 5 years to obtain current information. Thus, the period considered was from 2017 to 2022.

## METHOD

### Record

This study was prepared following the recommendations of the PRISMA statement for systematic reviews^(9)^. The protocol was registered in the Research Project Capture and Monitoring System of the Autonomous University of Baja California, Mexico.

### Eligibility Criteria

Studies that met the following characteristics were included: 1) primary research studies from 2017 to 2022; 2) articles published in Spanish or English; 3) descriptive and correlational studies; and 4) migrant population. To obtain additional articles, the bibliographic references section was located, and the relevance of adding them to this review was analyzed. Studies that did not clearly report the prevalence of condom use and drug consumption were excluded, as well as studies that did not have an association analysis.

### Search Strategy

To locate the keywords, the Medline thesaurus in Spanish DeCS was used, and subsequently the Medline thesaurus in its English version, medical Subject Headings (MeSH), was searched. Among the terms used, the following are highlighted: “Condom use”, “drug consumption”, and “migrants”. Synonyms and spelling variants are considered, as well as the use of Boolean operators and quotes. The bibliographic search was carried out in the following databases: PubMed, EBSCO, WEB of SCIENCE, Elsevier, Scielo, Redalyc, both in English and Spanish. The use of filters, such as time periods and fulltext articles, was implemented. The terms were piloted in different electronic databases and the references were exported to the online Mendeley bibliographic software, for storage and elimination of duplicate articles. Only those published after 2017 were considered. The details of the search are specified in Chart 1.

**Chart 1:** Databases and their respective search strategy – Tijuana, BC, Mexico, 2022.

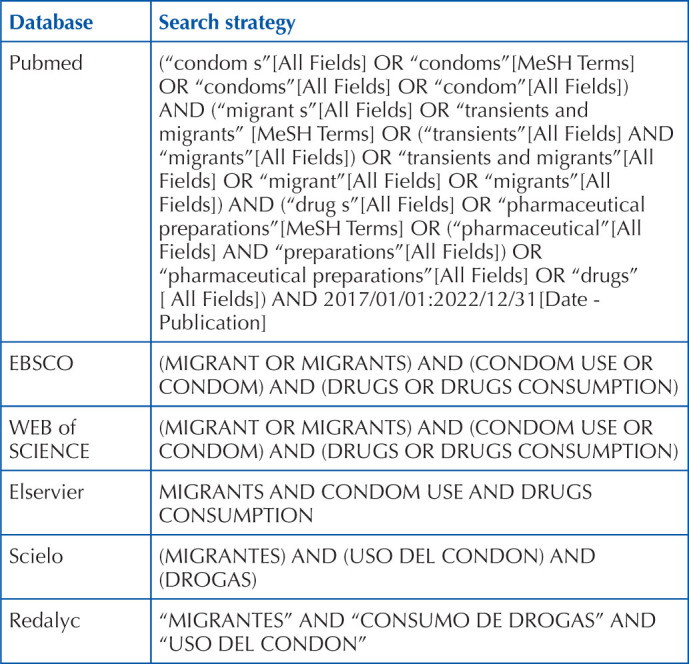

### Selection of Articles

**Figure 1:**
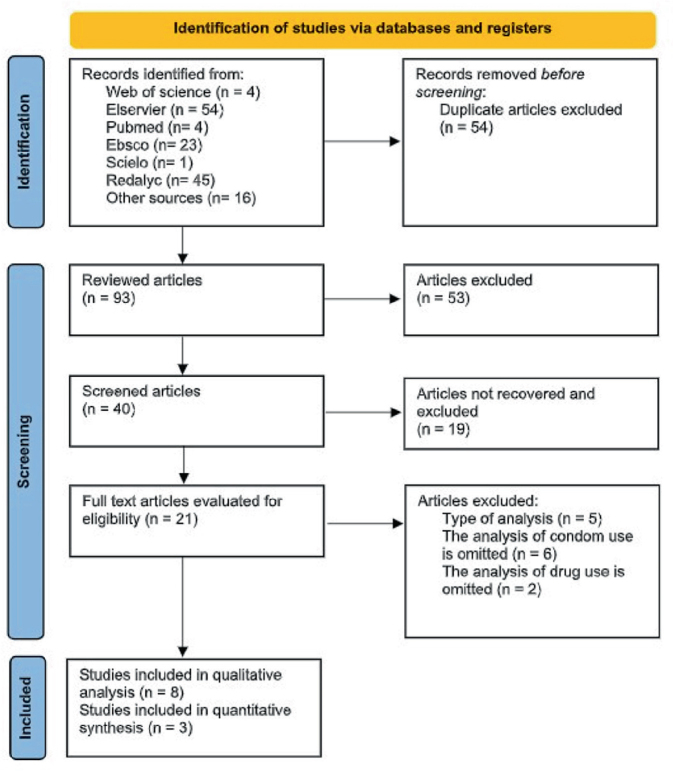
Diagram of the search strategy and selection of articles for systematic reviews PRISMA – Tijuana, BC, Mexico, 2022.

Once duplicates were eliminated, the titles of the documents found in the search were reviewed. Seven reviewers evaluated the relevance and pertinence of the articles content through STROBE, for the construction of the synthesis, and thereby the evaluation of the risk of bias. After this, they proceeded with the abstracts to find studies that met the inclusion criteria. The PRISMA flowchart is shown in Figure 1 ^(9)^, displaying the process of selection and exclusion of articles.

## EXTRACTED DATA

The data extracted from the selected articles were the following: authors, year of publication, journal, sample size, place where the study was carried out, instruments implemented, results obtained both in the use of condoms and in drug consumption, the association between them, and variables that were found to be significant with the study variables.

## RESULTS

The search strategy found 147 articles with the combination of terms and other sources. They were imported into the Mendeley bibliographic manager, where duplicates were removed. First, the studies titles and abstracts were reviewed; however, there were still duplicate articles that were not recognized by the administrator, since there were variations in the authors’ names and in the years of publication. These were manually excluded. A total of 53 articles were excluded, based on the title, abstract, and identification of duplicates due to variations in the authors’ names. In the second screening, 13 articles were excluded due to the type of statistical analysis, omission of the analysis on drug consumption, and/or condom use. Sample sizes ranged from 192 to 35,841 participants. The total number of participants was 56,483.

The selected studies were carried out in Germany^(10)^, China^(11)^, Spain^(12)^, USA^(13)^, Mexico^(14,15)^, Thailand^(16)^ and India^(17)^. All studies examined migrant populations; however, some studies were even more specific regarding population, such as migrant farm workers^(14)^, male migrants who have sex with men^(11)^, migrant sex workers^(16)^.

The instruments used to measure condom use were: sexual partner tracking questionnaire^(14)^; modified HIV knowledge questionnaire^(15)^; model questionnaire for drug-related infectious diseases^(10)^; face-to-face interview about sexual and risky behaviors^(16)^; structured interview including sexual practices; frequency of condom use and condom use in the last sexual relationship^(11)^; sociodemographic characteristics questionnaire that included risky sexual behaviors^(12)^; condom use resistance survey^(13)^; the Migrant Service Provision System (MSDS), in which risk profiles and health-seeking behavior are reported^(17)^. Besides exploring condom use, some studies performed serological tests to identify STIs^(10–13,15,16)^. It should be mentioned that the prevalence of condom use in the study population was relatively low in all the studies analyzed, and that they report inconsistent condom use. The details are listed in Chart 2.

**Chart 2:** Prevalence of condom use and drug consumption in migrants – Tijuana, BC, Mexico, 2022.

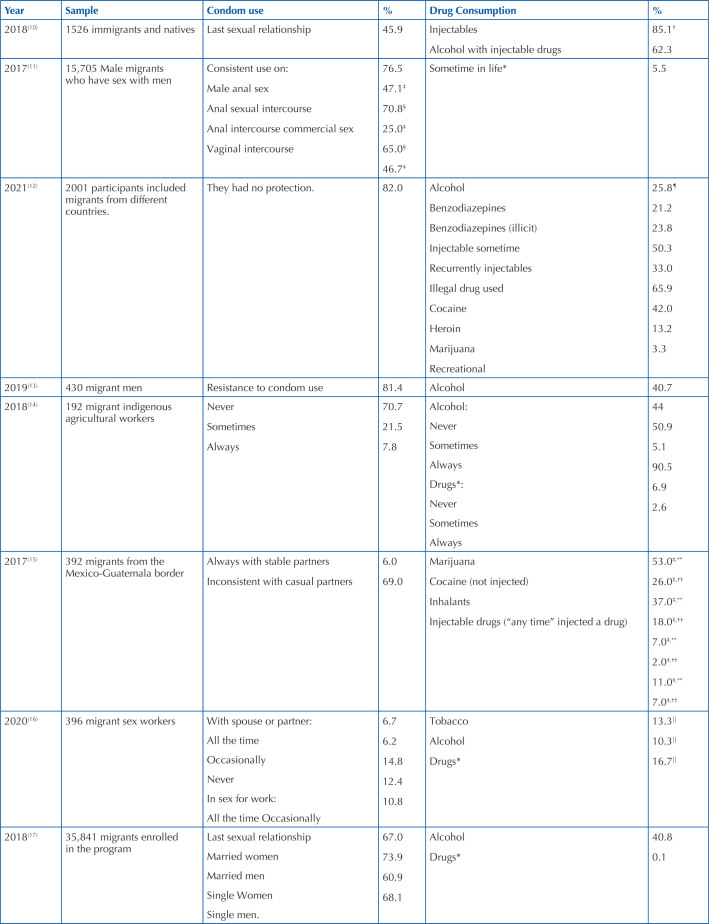

* Types of drugs not described; ^†^Last 30 days; ^‡^Last 6 months; ^§^Last year; ^||^Last 3 months; ^¶^Every day; ^**^Men only; ^††^Women only.

On the other hand, the instruments to evaluate drug consumption were the following: Sexual partner tracking questionnaire, which contains a section on alcohol and illegal drug use during sex^(14)^; interview about substance use^(15)^; injection drug use^(10)^; face-to-face interview with health history including drug use^(16)^; history of injection drugs with dichotomous response^(11)^; sociodemographic characteristics questionnaire that included drug consumption^(12)^; alcohol consumption for three months^(13)^; and MSDS^(17)^. The drugs commonly used by the study population were alcohol, marijuana, cocaine, inhalants, and opioids.

Chart 1 presents the prevalence of condom use and drug consumption, which were extracted from the eight articles that met the inclusion criteria. It is worth mentioning that there is a notable difference in sample size. Regarding the results of the prevalence of condom use, these differ in the results reported in the studies analyzed, since some report high^(10,11,17)^ and others low^(12–16)^ prevalence. Studies show that migrants used a condom in their last sexual relationship with percentages ranging between 45 to 74%^(10,11,17)^. Regarding the prevalences reported as relatively low, the percentage of constant use of condoms in sexual intercourses ranged between 6 and 8%^(14–16)^.

In contrast to reports of inconsistent use or no use of condoms, prevalence ranged between 69 and 82%^(12–15)^. Some of the reasons for not using this barrier method with casual partners, mentioned by both men and women, were that they “didn’t want to” or “didn’t like to wear them” (52% of women, 49% of men); and because “[the partner] seemed healthy” (18% of men and 28% of women)^(15)^. The ways in which condom use was reported was different in all the studies analyzed, ranging from always or never^(14,15)^, last sexual relationship^(10,17)^, consistent^(11)^ and inconsistent^(15)^, also addressing resistance to condom use^(13)^.

Similarly, the results found regarding drug consumption also present differences in prevalence and in the drugs studied, since some report the consumption of alcohol^(13,14,16,17)^, tobacco^(16)^, injectable drugs^(10,12,15)^, the combination of some of them^(10)^, inhalants^(15)^, but there are studies that do not specify what drugs the participants use^(11,14,17)^. In the case of studies reporting alcohol consumption, they show a prevalence that ranges between 5.1 to 40.8%^(11,13,14,16,17)^.. Two studies presented similar prevalences^(13,17)^. Tobacco consumption occurred in 13.3%^(16)^, unlike 53% of users who consume marijuana^(15)^. The injectable drugs that were reported in the studies included cocaine, buprenorphine, diacetylmorphine, diacetylmorphine, fentanyl, heroin, methadone, polamidon, tramadol, and tilidine^(10,12)^, and the combination of these with alcohol was also reported^(10)^.

Chart 3 presents the probabilities or associations between the use of condom and drug consumption. This was extracted from the eight articles that met the inclusion criteria. It is necessary to mention that the results closest to the operational definitions of the study are presented; therefore, the analyzed variables are specified.

**Chart 3:** Associations between condom use and drug consumption in migrants – Tijuana, BC, Mexico.

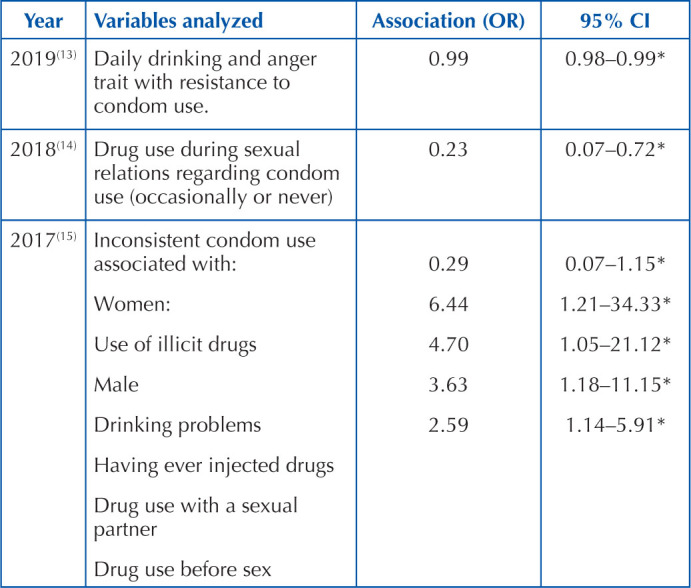

* p < .05.

In the analysis corresponding to condom use with drug use during sexual relations, the probability that a migrant who uses drugs during sexual relations uses a condom occasionally or never is 0.23 times greater than for those who do not use drugs^(14)^. Similar results were found in the case of inconsistent condom use in migrant women who use illicit drugs, who have a 0.29 greater probability of using condoms inconsistently, compared to those who do not use them^(15)^.

On the other hand, men who consume alcohol have a 6.44% greater risk of using condoms inconsistently^(15)^. Men who have injected drugs at some time in their life have a 4.70 higher risk of inconsistent condom use^(15)^. Men who use drugs with their sexual partner have a 3.63 higher risk of inconsistent condom use^(15)^. Men who use drugs before sex have a 2.59 higher risk of inconsistent condom use(15). All these cases are in comparison with men who do not use drugs^(15)^. In another study, it was found that the consumption of alcoholic beverages in migrants with the psychological trait of anger was associated with resistance to condom use^(13)^.

## DISCUSSION

This review allowed us to estimate both condom use and drug consumption in migrants, also allowing us to identify the association between these variables. It shows a statistically significant association, although the way of evaluating both variables studied is not consistent.

The results of the prevalence of condom use were consistent and similar in most studies analyzed; however, the difference between other studies is notable, which may be due to the geographical area where they were carried out, or due to cultural differences, to conditions or situations in which they live at that particular moment, since some are migrants in transit, or established, others are temporary workers who return to their place of origin or are dedicated to commercial sex. Although the population in all the studies was of migrants, the population is even more specific; therefore, it cannot be stated that these results can be representative of the migrant population in general.

Although some reviews have focused on the study of the prevalence of STIs^(7,18)^, there are data such as the prevalence reported as low in this study^(14–16)^ that are consistent with that reported in a review study^(7)^. The instruments implemented in the studies analyzed were totally different; therefore, it is not possible to definitively determine the prevalence of both condom use and drug consumption. The use of instruments that are highly recognized and used by UNAIDS/UNAIDS is suggested.

Regarding the places where the data were collected, all were carried out at strategic points where they could be easily found or in health services where they go for health care. This is consistent with what was reported in a review^(18)^ mentioning that closed places are ideal for data collection and interventions.

Among the limitations of the study, the scarce information on the topic in the migrant population was identified. Following the analysis, the measure used by the studies for the variable is considered a limitation, since the instruments were not homogeneous. Likewise, in the literature considered, the study of the variables was carried out separately, which did not allow us to see the relationship or association among them, so the number of selected studies was reduced even further.

Regarding public and community health, this is a great challenge for nursing, as part of the health system. It can contribute to the reduction of risky sexual behaviors, and to the reduction of the consumption of substances that are harmful to their health, in the first instance, through scientific research that allows establishing the relationship or interaction of variables involved in this type of behavior. This way, it is possible to identify dimensions of knowledge that can contribute to the foundation of educational or behavioral interventions that are effective for this population, through sexual health promotion and education, thereby supporting the prevention of HIV/AIDS in this population.

Establishing a health education program for people who migrate, which includes the provision of condoms, rapid HIV testing, and referral of those who test positive, can help reduce the risk of acquiring HIV.

## CONCLUSION

This review allowed identifying variations in the prevalence of condom use and drug consumption in the migrant population. The findings suggest the homogenization of the way of evaluating the study variables and the expansion of interventions in the community to promote access to sexual health services, particularly in Mexico, which is a transit country for migrants, and where these services have no cost. There is an association between condom use and drug consumption, which was statistically significant. Drug consumption favors inconsistent condom use, increasing the risk of acquiring an STI, and can lead to other mental health issues derived from the use of these substances.
